# Obstetrics

**Published:** 1900-06

**Authors:** Walter C. Swayne


### OBSTETRICS.

**The use of the intra-uterine douche.**—E. P. Davis[1] describes the chief dangers to be apprehended from intra-uterine douching as being, firstly, the risk of septic infection being induced by the douche; and, secondly, the risk of hemorrhage from detachment of thrombi in the uterine sinuses.

But the indications for its use are also just these same two conditions—namely, hemorrhage and sepsis. In septic conditions its use is based on the sound principle of removing retained and possibly decomposing clot, membrane, placenta, etc., from the uterine cavity by means of a stream of sterile fluid.

He advocates the use of sterile salt solution in preference to carbolic acid or other phenyl derivatives in cases where much depression is present and loss of blood has occurred, and considers that mercurial solutions should never be used in the uterus; he prefers to use the douche curette without anæsthesia, both for exploration and cleansing purposes, since he does not consider the finger long enough for the purpose, and uses a tube which is apparently a modification of Budin's. With regard to the use of the intra-uterine douche in hemorrhage, he remarks that it is not easy to maintain asepsis in these cases, and very rightly emphasises the necessity of having every preparation made in case of bleeding occurring, so as to minimise the risk of infection as much as possible. He also recommends the use of the iodoform or sterile gauze-tampon as an adjuvant after the use of the douche for hemorrhage. If retention of a portion of placenta or membrane is suspected, he advises the use of the douche curette for its removal should hemorrhage supervene, this being a fertile cause of secondary *post-partum* bleeding.

The value of intra-uterine douching has been brought to my own notice through the records of the Maternity Department of the Bristol Royal Infirmary. We have there for years been in

[1] *Am. Gynæc. & Obst. J.*, 1900, xvi. 196 (discussion), 272.

the habit of using an intra-uterine mercurial douche in every case where any instrument or the hand had been introduced into the uterine cavity for any purpose whatsoever. So far, in only one of a large number of such cases has any septic trouble occurred; while in the remaining cases in which no interference has taken place, in many of which no vaginal examination was made, the proportion of septic cases is much higher.

Mercurial poisoning is certainly a risk, but when we consider that the total amount of the salt in a quart of solution (1 in 2000) is 10 grains (about), and that to exceed the medicinal dose by the mouth it is necessary that half-an-ounce of solution should be completely absorbed, it strikes one that the danger can be exaggerated. I have met with one case in which salivation occurred after the persistent use of intra-uterine mercurial douches; this was in the early days of its use as an antiseptic in midwifery, when the necessary precautions were not well understood. The danger of mercurial poisoning following the use of a single douche of mercurial salt is infinitesimal.

The preparation of a hot douche in all cases, with sterilisation of the tube and every preliminary precaution, will not be lost on any one who has had to treat a case of real *post-partum* flooding. A case is recorded in which a Continental practitioner allowed his patient to bleed to death while making preparations to secure asepsis. The danger in severe hemorrhage is one that admits of no delay; a rush of blood forming a stream apparently nearly as thick as the wrist, and shooting out from the patient's vagina two or three feet into the room, is not going to allow any time for precautions to avoid sepsis if the patient's life is to be saved. All precautions should be taken beforehand. The above is a personal experience which I hope never will be repeated, although the patient was successfully steered between the Scylla of death from hemorrhage and the Charybdis of septic infection.

Intra-uterine douching should be reserved for instrumental and operative cases, for cases of hemorrhage, and for cases of suspected septic infection with high temperature; the solution used is not important, but it must be sterile; in septic cases, if its use is followed by a rise of temperature, exploration of the uterus should follow. The intra-uterine douche is perfectly safe if used intelligently and with proper precautions, and has proved invaluable on more than one occasion in cases under my care, when either *post-partum* hemorrhage occurred or where septic infection, proved bacteriologically afterwards, was suspected at the time of its use.

The **treatment of septicæmia** by anti-streptococcic serum is a subject which has lately become of great importance, and although at present the indications for treatment, owing to the difficulty of making an exact bacteriological diagnosis in time for the treatment to be successful, are not very clear, yet the increasing record of successful cases makes us hope that eventually this method will be reduced to an exact one and placed on a footing more secure than the empirical position it now occupies. Herman [1] records two cases in which life appears to have been saved by the use of the serum.

Considerable divergence of opinion was evident during the discussion of Dr. Herman's cases, some condemning the use of the serum altogether except in cases where the existence of the streptococcus was definitely proved; the general consensus was that its use should not be allowed to exclude the application of more radical measures. Seigneux [2] reports a case in which a primipara of 21 was attacked with acute sepsis at the close of pregnancy, which did not improve on her delivery a few days later. A subcutaneous injection of 600 c.c. of salt solution was given without benefit, but after the injection of 20 c.c. of Marmorek's serum her symptoms abated and she gradually recovered.

A committee of the American Gynecological Society [3] have lately been engaged in the investigation of the results of serum treatment, and made a collection of 354 cases treated in this way, the analysis of which gave some startling results.

The cases collected were obtained as follows:—From France 214, Germany 15, Great Britain and America 125.

Of the collected cases 72, or 20.6 per cent., resulted fatally; but of those cases in which the streptococcus was demonstrated the mortality was 33 per cent., and the committee doubted whether the natural mortality was as high as this, and quoted Fischer, who in 60 cases of pyæmia met with 32 deaths, 19 recoveries, and 9 not traced (this mortality, it should be noted, is one of 53 per cent.).

Whitridge Williams, Pryor, and Fry gave details of their own special treatment of these cases. Whitridge Williams gives a single intra-uterine douche of sterile salt solution after a culture has been taken from the uterine cavity, and relies on large doses of strychnia and alcohol. Pryor opens the posterior *cul de sac*, plugs the pelvis tightly with iodoform gauze, and gives large injections of normal saline, both subcutaneously and by the rectum. Fry, after collecting the discharge from the uterus for cultivation, uses the intra-uterine douche until the bacteriological diagnosis is made, and then stops the local treatment and administers the serum in large doses. The conclusion of the committee was that Marmorek's serum does *not* cure streptococcus infection. Spencer, [4] in a paper read at the meeting of the British Medical Association, quoted the above statistics and opinions; he considers the use of the serum as unscientific and superficial, and comes to the conclusion that it is useless.

[1] *Tr. Obst. Soc. Lond.*, 1899, xli. 346.

[2] *Centralbl. f. Gynäk.*, 1899, xxiii. 1489; abstract in *Brit. Gynæc. J.*, 1899–1900, xv. 645.

[3] *Phila. M. J.*, 1899, iii. 1199; abstract in *Brit. M. J.*, 1899, i. Epitome, p. 94.

[4] *Brit. M. J.*, 1899, ii. 965.

After reviewing the various opinions, one is inclined to conclude with their supporters that the serum is of little use: before doing this a few facts should be borne in mind, and any one who cares to take the trouble can easily gain the information for himself. Taking Bristol alone, the mortality from puerperal fever is between 60 and 70 per cent. of the notified cases. A reference to the Medical Officer of Health's report will easily verify the correctness of this; I am quoting from memory, as I have not verified the figures lately,—at any rate, it is far in excess of 33 per cent. No doubt it will be objected that this includes all cases, not including streptococcus infections only. That of course is obvious, and does not in reality detract from the importance of the figures. Another point is, that the serum is only used in the more acute cases; *e.g.*, a culture taken from the uterus of a woman who had developed a high temperature was pronounced to be pure streptococcus of a most virulent type; but she recovered after a single intra-uterine douche. (The case is recorded in the books of the Bristol Royal Infirmary.) This case would have been passed over had it not been for the culture.

Further, it is noteworthy that cultures from the uterus, even in bad cases, are nearly always sterile if one intra-uterine douche has been given: this proves nothing, as in one such case in which I removed a small piece of placenta streptococci grew in profusion from the uterine surface of the placenta after removal, although it had been drenched with corrosive sublimate solution, 1—2000, during the process of removal. Therefore a sterile culture from the uterine cavity does not necessarily prove that a streptococcic infection is not present. Another point that has struck me is the difference in the mortality rate between cases treated early and those treated late. In many cases the invasion can be abated by prompt local treatment; but these may be none the less streptococcic infections: when several hours have elapsed before treatment the mortality is enormously increased.

Waiting for bacteriological diagnosis means a lapse of thirty hours at least. If the patient has a streptococcic infection, her chance is about halved by this, even although local measures have been adopted. Therefore, if the serum is to have a chance of doing any good, it must be used at once, without waiting for bacteriological diagnosis. It seems doubtful whether it is harmful; and although we cannot use it at present except empirically, we shall only do our patients harm by making each case one for strictly scientific investigation. Personally my experience has been that it is not harmful, and though not always successful in saving life, it has, as a rule, caused amelioration of the symptoms, checking headache, reducing fever, and improving, at any rate temporarily, the patient's condition.

In one case in which it was used in conjunction with the intra-uterine douche alone, the effects were most striking, the temperature falling markedly after each injection, and ceasing to rise after the third injection, the patient making a good recovery.

Ahlfeld[1] in puerperal fever and protracted difficult labour, of which he records 80 cases of the two together when infection was suspected, has used intra-uterine injections of alcohol of a strength of 50 per cent. with good results, fever disappearing after one or two injections, and the patients escaping any serious uterine affection. In Switzerland out of 2900 deaths in childbed, 1536 were due to puerperal fever, or 53 per cent. This forms rather a striking commentary on the mortality of 20.6 per cent. in the cases recorded by the American committee after serum treatment, and also on the mortality of 33 per cent. in the cases where streptococcus infection was proved and the serum used.

WALTER C. SWAYNE.

